# Prenatal and birth factors associated with child autism diagnosis: a birth cohort perspective

**DOI:** 10.1038/s41390-025-04747-x

**Published:** 2026-01-15

**Authors:** Lada Holland, Katherine Drummond, Sarah Thomson, Luba Sominsky, Wolfgang Marx, Chloe Love, Samantha L. Dawson, Leonard C. Harrison, Richard Saffery, Christos Symeonides, Mimi LK Tang, David Burgner, Peter D. Sly, Peter Vuillermin, Anne-Louise Ponsonby, the BIS Investigator group, Toby Mansell, Sarath Ranganathan, Martin O’Hely

**Affiliations:** 1 The Florey Institute of Neuroscience and Mental Health, The University of Melbourne, Parkville, VIC Australia; 2 Child Health Research Unit, Barwon Health, Geelong, VIC Australia; 3 Institute of Mental and Physical Health and Clinical Translation, School of Medicine, Deakin University, Geelong, VIC Australia; 4 Murdoch Children’s Research Institute, Parkville, VIC Australia; 5 Walter and Eliza Hall Institute of Medical Research, Melbourne, VIC Australia; 6 Centre of Community Child Health, Royal Children’s Hospital, Parkville, VIC Australia; 7 Minderoo Foundation, Perth, Australia; 8 Department of Paediatrics, University of Melbourne, Parkville, VIC Australia; 9 Children’s Health and Environment Program, Child Health Research Centre, The University of Queensland, South Brisbane, QLD Australia

## Abstract

**Background:**

Autism spectrum disorder (autism) describes a heterogeneous neurodevelopmental phenotype arising from the interplay of environmental and genetic factors in early life.

**Methods:**

In a general population birth cohort, we employed a scoping approach to identify prospective associations between prenatal and birth factors and a subsequent autism diagnosis.

**Results:**

Factors associated with increased likelihood of autism included those related to i) maternal health (maternal pre-pregnancy body mass index, pre-existing maternal mental health conditions, maternal use of selective serotonin reuptake inhibitors) ii) environmental exposures (maternal passive tobacco smoke exposure, and exposure to vinyl floors) iii) demographic factors (socioeconomic disadvantage). Factors associated with a decreased likelihood of autism included maternal dietary nutrition and supplementation (higher folic acid, magnesium, and iron, as well as adherence to the Australian Dietary Guidelines).

**Conclusion:**

Our findings extend the evidence that autism may have a multifactorial origin in early life. Further studies should explore the composite effects of these prenatal and birth factors on autism outcomes via shared biological pathways, such as inflammation, and oxidative stress, in concert with genetic predisposition.

**Impact:**

- Autism spectrum disorder (autism) is a multifactorial condition. Here we report on multiple prenatal environmental, demographic, maternal and pregnancy factors that are associated with an increased likelihood of an autism diagnosis. For example, adherence to the Australian Dietary Guidelines during pregnancy is linked to a reduced likelihood of autism in the offspring, consistent with mounting evidence that prenatal nutrition impacts brain development. We examine how the multiple risk factors, identified by our comprehensive approach, may be linked to shared biological mechanisms. Future work should examine composite exposure measures acting through shared mechanisms as a more productive approach to understanding aetiology than focusing solely on individual exposures.

## Introduction

In this paper, we use the term *‘autism’* rather than *‘autism spectrum disorder’*, in keeping with the preferences expressed by the autism community.^1,2^ Autism is a neurodevelopmental condition that influences how a person interacts and communicates with others and experiences the world around them.^3^ Autism diagnostic criteria require that the individual has persistent challenges in social interaction and communication, along with restricted, repetitive, and inflexible behaviours, interests, or activities that are atypical for the individual’s age and context.^4^ The World Health Organisation reports that the global prevalence of diagnosed autism is about 1 in 100 children worldwide. However, this estimate varies across studies and countries.^5^ In the Barwon Infant Study (BIS) cohort, the estimated prevalence of autism in the Barwon region, Victoria, Australia, was 74.8 per 1,000 children up to 11.5 years of age.^6^ Several countries are reporting an increase in autism prevalence over time. A multinational comparison study reported that the 10-year cumulative prevalence of autism (1990 to 2000) increased by 96% in Finland and by 354% in Denmark.^7^ In the US, the prevalence of autism diagnosis increased by 50% (from 18.5 per 1,000 to 27.6 per 1,000 children) between 2016 and 2020.^8^ Whether there has been a true increase in autism incidence remains controversial;^9^ nonetheless, the role of modern environmental factors requires careful evaluation.

The aetiology of autism is complex and likely heterogenous, involving multiple factors and gene-environment interactions, which dynamically influence risk and development over time. Twin studies indicate that genetic factors account for approximately 30–40% of autism risk.^10^ Multiple environmental factors impact gene expression via epigenetics and other mechanisms. Shared underlying biological mechanisms are not fully understood, but those of particular emerging interest include inflammation,^11^ mitochondrial dysfunction,^12,13^ and oxidative stress.^14^

In recent years, studies have focused on a range of prenatal and birth factors associated with autism.^15–17^ Reported prenatal factors linked to increased risk of autism include maternal age, mental health conditions, obesity, gestational diabetes, hypertension, infections during pregnancy, and maternal medication use such as selective serotonin reuptake inhibitors (SSRIs)^16–18^ and sodium valproate.^19^ Birth factors have also been linked to autism, including pregnancy and labour complications, delivery by caesarean section, breech presentation, preterm birth, foetal distress during birth, low birth weight, birth asphyxia.^16^

For many of these factors, findings vary between cohorts, possibly because autism is a multifactorial phenotype with a small individual effect size, and contributing factors can differ from population to population. Further, some studies lack power or have considered these factors in isolation.^16^ Focusing on a single or a small number of factors can limit the inference across a multiple risk factor range and can overlook how these factors may interact or contribute collectively to autism development.

In an Australian birth cohort, the BIS, we have demonstrated that the composite effect of multiple risk factors is important to consider, particularly if the risk factors are operating through a shared pathway.^20,21^ For example, oxidative stress, which occurs when the production of reactive oxygen species exceeds antioxidant and repair capacity, which is implicated in autism. We demonstrated that composite exposure to early life risk factors was associated with substantially higher odds of autism than that of any single exposure.^22^ Here, we aim to employ a scoping approach to examine the prospective associations between a wide range of prenatal and birth factors quantified in BIS and subsequent autism diagnosis, with the goal of identifying key factors of interest that may warrant attention for future causal analyses, including investigation of combined effects via shared biological pathways such as oxidative stress and inflammation. We extend our previous report on early autism symptom risk factors^17^ by examining a wide range of factors across various domains, such as demographic and household factors, maternal health and diet, environmental pollutants, and birth factors and autism diagnosis in children. We then aim to interpret the findings with consideration of the multifactorial aetiology of autism and consider the extent to which the risk factors here may be operating through common causal pathways. Using VOSviewer literature network analysis, we examined the extent to which the potential risk factors identified may be operating through common causal pathways.

## Methods

### Study design and participants

A population-derived birth cohort (mothers, *n* = 1064; infants, *n* = 1074, including 10 set of twins) was recruited using an unselected antenatal sampling frame in the Barwon region of Victoria, Australia. The recruitment for this longitudinal cohort was between June 2010 and June 2013. Eligibility criteria, population characteristics and measurement details have been previously described.^23^ The BIS cohort protocol was approved by the Barwon Health Human Research Ethics Committee (HREC 10/24) and the participating families provided written informed consent.

### Exposure measures

Over 100 prenatal and birth factors were examined and listed in supplementary data (Box S1). The main domains examined were demographic and household factors, maternal health (including physical and mental) and diet, maternal exposure to environmental pollutants in pregnancy, and birth factors. Literature and data driven approaches were used to prioritise this final report. A data-adaptive approach is useful in low-knowledge settings where the list of important factors may be incomplete.^24^ We had an emphasis on prenatal dietary nutritional factors to reflect the quality and nutrient density of prenatal diet, given their known roles in brain development, including neurotransmitter signalling^25^ and neuroprotective effects,^26^ as well as autism development.^27^ During the in-clinic appointment at 28 weeks gestation, information on preconception and antenatal period was collected, including: demographic and household composition, antenatal health, nutrition and dietary patterns, vitamin intake, and supplementation. Additional information on 3^rd^ trimester exposures such as fever, vitamin use and supplementation, alcohol use, smoking and passive tobacco exposure was self-reported retrospectively (at 4 weeks after birth) using a self-reported questionnaire. Information regarding medical conditions, including psychosocial conditions (anxiety, depression, bipolar or other), and delivery information was obtained from antenatal medical records. The selection of derived variables and categorisation were based on prior literature and is further described in detailed in Supplementary Methods. The scope of this study does not include genetic measures or other exposures measured from biological samples, such as urinary or blood chemicals, metabolomic and lipidomic measurements.

### Literature-based evidence mapping and contextualisation

A literature search was conducted to review known associations between prenatal and birth factors and autism (Table S1). These factors were further contextualised in relation to shared biological pathways such as oxidative stress and inflammation (Table S1). We classified the strength of prior evidence using a ranking system based on the hierarchy of evidence (systematic reviews^28,29^ or large cohort studies.^30^) Findings from this study then informed a VOSviewer literature network analysis of clusters by keywords (Box S2).

### Verified autism diagnosis

The process for obtaining a child’s verified autism diagnosis was previously described in detail.^6,31^ In brief, during a telephone interview in 2020–2022, 868 parents reported on their child’s autism status, with 80 parents reporting that their child had received a diagnosis or was under investigation for autism. Medical records were reviewed for 79/80 children; 64 children were confirmed to have a diagnosis of autism made by their treating paediatrician against the DSM-5 criteria. Twelve children were excluded because they were lost to follow-up, their diagnosis was still under investigation; they had an inconclusive diagnosis; or were not reported by their parent as diagnosed with autism but had a clinician-confirmed diagnosis at the time of verification completion (31 October 2023). Eight participants whose parents reported their child as under investigation for autism at the 2020–2022 review did not receive a subsequent diagnosis. Further, the verified autism diagnosis was compared to the Strengths and Difficulties Questionnaire (SDQ) scale available in BIS ages 4–10 with many subscales (Conduct Problems, Emotional Symptoms, Hyperactivity Score, Peer Problems, Prosocial Behaviour, Total Difficulties) being significantly associated (*p*<0.001). Detailed description of the diagnostic verification process including comparisons to other neurodevelopment outcomes is described elsewhere.^6^

### Statistical analysis

Plots and summary statistics were used to assess the distribution of prenatal and birth factors. We calculated correlations between prenatal and birth factors and child’s autism. Visual representation of the correlations was generated using statistical package “corrplot” in R version 4.3.2 R Core Team, 2024 (Fig. 2, Fig. S1). Separate multiple logistic regression models were used to assess associations between each exposure and the binary autism outcome after adjusting for child’s sex and child’s age when autism status was first reported. These processing factors were applicable across all exposures.

Additionally, no adjustments for multiple comparisons were undertaken in this study based on the following rationale: First, factors are not independent of each other (Fig. 2, Fig. S1) and may be acting jointly. Secondly, for exploratory analysis, it is not necessary to adjust for *p*-value.^32–35^ Thirdly, it has been argued that corrections for multiple comparison may also reduce power, increasing the risk of type II error.^7^ Given the nature of this study, we prioritised detection, that is, avoiding type II errors rather than avoiding type I errors. The focus was on identifying potential associations for future research. Further, we followed suggestions by Althouse (2016) to (1) describe what was done in our study; (2) report effect sizes, confidence intervals, and *p* values without imposing a false dichotomy (e.g., interpreting *p values* <*0.05* as ‘significant’); and (3) let readers judge the relative weight of the conclusions.^32^ Data was analysed using STATA version 17 (StataCorp LP). Further sparse bias was tested for variables with low cases (Supplementary Results).

## Results

### Cohort summary

#### Demographic and household composition

**Fig. 1:**
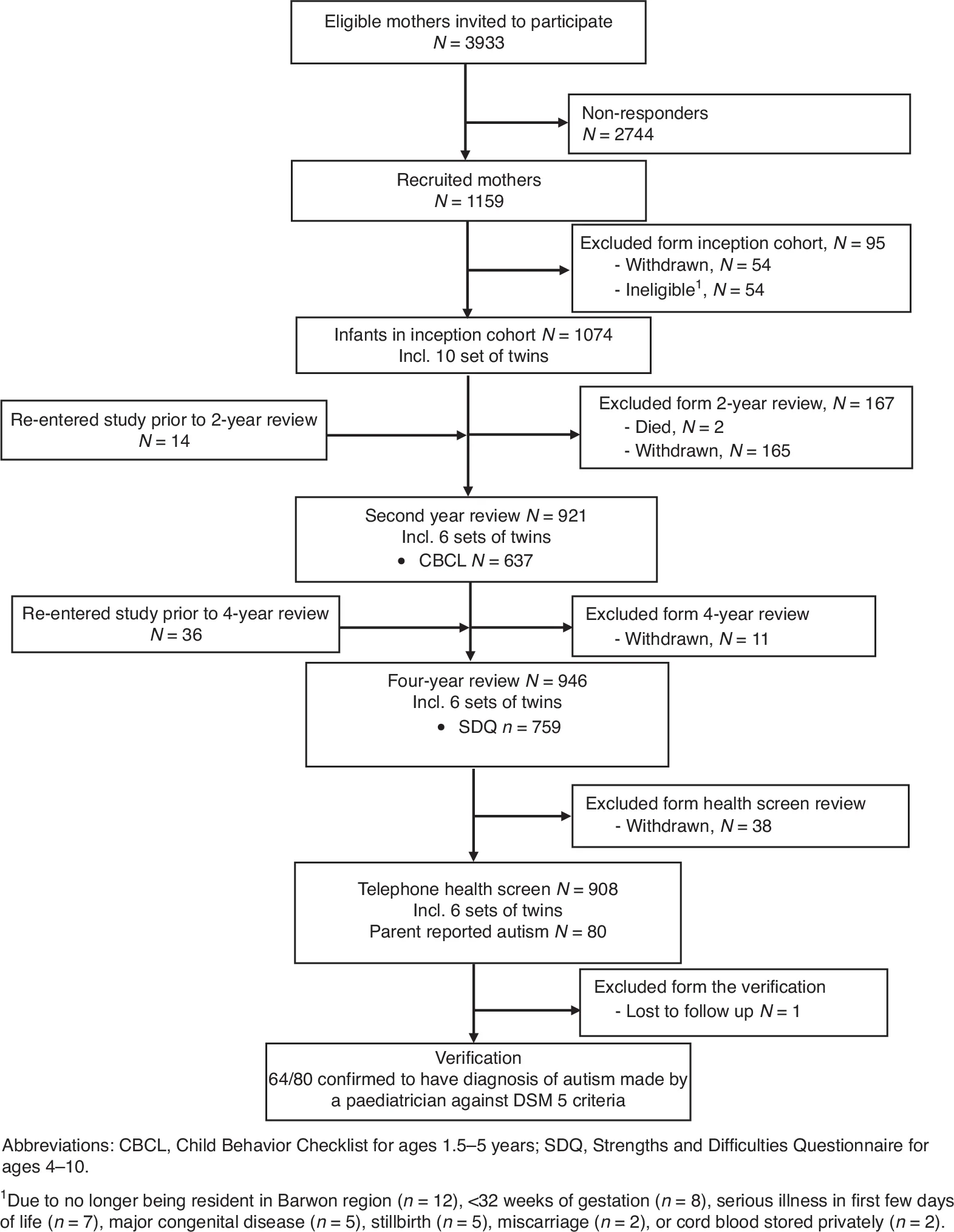
Barwon Infant Study participant flowchart. CBCL Child Behavior Checklist for ages 1.5–5 years, SDQ Strengths and Difficulties Questionnaire for ages 4–10.^1^ Due to no longer being resident in Barwon region (*n* = 12), <32 weeks of gestation (*n* = 8), serious illness in first few days of life (*n* = 7), major congenital disease (*n* = 5), stillbirth (*n* = 5), miscarriage (*n* = 2), or cord blood stored privately (*n* = 2).

Participant demographics for the inception cohort are shown in Table S3 and study flowchart is shown in Fig. 1. In the analysis sample, the mean [SD] age of children at completion of the health screen was 9.0 [SD = 0.7] years (Table 1). The mean age at the completion of the autism verification was 11.5 [SD = 0.8] years. Of the 64 children diagnosed with autism, 43 were male (67.2%) and 21 were female (32.8%). In the analysis sample (*n* = 856), the average age of mothers and fathers at conception was 31.8 [SD = 4.5] years and 33.7 [SD = 5.5] years respectively (Table 1). Fifty-six per cent (*n* = 478) of mothers and 37.3% (*n* = 312) of fathers had a university education (undergraduate or postgraduate). A total of 74.1% (*n* = 628) of participants had all grandparents of European descent.

**Table 1:** Prenatal and birth factors and child autism diagnosis.

Factors of interest		Child’s autism diagnosis^1^				
		% (*N*), Mean [SD](N) or *median [IQR] (N)*		AOR	95% CI	*P*-value
**Demographic and household composition**						
Child sex assigned at birth:						
Female		48.2% (413)		Reference		
Male		51.8% (443)		**2.01**	**1.17, 3.44**	**0.011**
Mother’s age at conception (years)		31.8 [4.5] (856)		0.98	0.92, 1.03	0.401
Father’s age at conception (years)		33.7 [5.5] (814)		0.98	0.93, 1.03	0.342
Mother university educated: yes^a^		56.0% (478)		**0.59**	**0.35, 0.98**	**0.043**
Father university educated: yes^a^		37.3% (312)		0.81	0.47, 1.42	0.468
Household income AUS$100, 000: yes^b^		53.4% (447)		1.35	0.78, 2.35	0.285
SEIFA (continuous per 100 points)^c^		10.2 [0.8] (847)		**0.72**	**0.54, 0.95**	**0.019**
Birth order^d^:						
First		44.8% (383)		Reference		
Second or later		55.2% (472)		**0.56**	**0.34, 0.95**	**0.031**
Parity – total siblings with same mother						
0		44.8% (383)		Reference		
1		34.9% (298)		**0.42**	**0.22, 0.81**	**0.01**
2		16.5% (141)		0.81	0.4, 1.64	0.551
3+		3.9% (33)		0.88	0.26, 3.06	0.845
*trend*						*0.301*
Single parent^e^		2.8% (24)		**3.62**	**1.29, 10.17**	**0.015**
All grandparents are of European descent		74.1% (628)		0.94	0.53, 1.69	0.844
**Antenatal health**						
Maternal pre-pregnancy BMI (kg/m^2^)		25.4 [5.5] (755)		**1.06**	**1.02, 1.11**	**0.005**
Maternal weight at 28-week review (kg)		80.7 [15.4] (693)		**1.02**	**1.003,1.04**	**0.022**
Weight gain during pregnancy >20 kg: yes		11.6% (61)		0.97	0.36, 2.59	0.949
Any alcohol intake during pregnancy: yes		428 (856)		0.72	0.43,1.21	0.211
Any smoking during pregnancy: yes		7% (60)		1.45	0.59, 3.52	0.417
Any recreational marijuana use during pregnancy: yes		1.3% (11)		**5.68**	**1.43, 22.62**	**0.014**
Perceived Stress Score, T1&2		18.1 [6.6] (647)		1.01	0.96,1.06	0.645
Edinburgh Depression Scale^f^:						
Low risk ( < 10)		84.6% (520)		Reference		
Moderate risk (10 to 12)		11.1% (68)		1.37	0.54, 3.48	0.506
High risk ( ≥ 13)		4.4% (27)		1.43	0.32, 6.44	0.64
Pre-existing psychosocial condition^g^		9.7% (83)		**3.93**	**2.1, 7.38**	**<0.001**
Any prescription medication use during T1&2: yes		44.0% (373)		**1.72**	**1.03, 2.88**	0.039*
Paracetamol use during T1&2: yes		79.3% (677)		1.76	0.85, 3.65	0.129
Antibiotic use during pregnancy: yes		16.9% (145)		0.97	0.49, 1.92	0.939
SSRI/SNRI use during pregnancy		4.2% (35)		**6.40**	**2.85, 14.35**	**<0.001**
SSRI/SNRI use during T1&2: yes		3.8% (32)		**6.43**	**2.76, 14.98**	<0.001*
SSRI/SNRI use during T3: yes		2.8% (24)		**9.01**	**3.70, 21.94**	<0.001*
Gestational diabetes mellitus: yes		4.7% (34)		1.22	0.36, 4.15	0.755
Preeclampsia: yes		3.4% (28)		2.27	0.75, 6.83	0.145
Hypertension: yes		2.4% (20)		1.37	0.38, 7.63	0.489
Prenatal adversity (score)^i^						
no adversity		51.2% (438)		Reference		
1		25.7% (220)		**2.04**	**1.09, 3.82**	**0.025**
2 and higher		23.1% (198)		**2.36**	**1.26, 4.42**	**0.008**
**Antenatal environmental factors and pollutants**						
Pesticide use during pregnancy		68.9% (580)		0.98	0.56, 1.71	0.945
Passive tobacco smoke prior conception, T1&2		10.6% (89)		**2.68**	**1.40, 5.12**	**0.003**
Ambient air pollution at birth:						
NO_2_ (ppb)		5.5 [2.0] (856)		1.07	0.95, 1.21	0.246
PM2.5 (µg/m^3^)		7.7 [0.9] (856)		1.02	0.77, 1.35	0.906
Heating in residence						
Central heating ducted		48.5% (266)		0.72	0.36,1.45	0.357
Reverse-cycle		29.7% (163)		1.29	0.62,2.67	0.491
Gas heating		25.5% (140)		1.94	0.95,3.95	0.067
Cooling in residence						
Fixed wall air conditioning unit		55.1% (302)		0.84	0.42,1.68	0.629
Ceiling fan		26.5% (145)		1.15	0.54,2.47	0.719
Portable fan		20.6% (113)		**2.15**	**1.03,4.48**	**0.042**
Signs of mould/dampness: yes		31.1% (170)		1.32	0.65,2.70	0.445
Vinyl flooring, living room: yes		4.6% (25)		**4.21**	**1.45,12.21**	0.008*
**Birth factors**						
Gestational age at birth (weeks)		39.4 [1.5] (856)		0.95	0.8, 1.12	0.517
Apgar score at 5 min		9.0 [0.8] (842)		0.84	0.65, 1.08	0.168
Labour duration (hours)		*5.0 [7.04] (855)*		1.01	0.97, 1.06	0.593
Preterm birth: yes (32–37 weeks vs rest)		4.3% (37)		**2.58**	**1.02, 6.51**	0.044*
Birth weight (in grams):						
<= 4200 g		91.0% (779)		Reference		
>4200 g		9.0% (77)		1.33	0.60, 2.93	0.478
Resuscitation required at birth^j^		0.8% (7)		2.17	0.25, 18.54	0.480
Childbirth weight (kg)		3.5 [0.5] (856)		0.92	0.56,1.5	0.736

**Bold** indicates an association with a *p* < 0.05;P-trend in italic, *due to low number of cases sparse bias was evaluated in supplementary results.

^1^Total cohort *N* = 856 which includes *N* = 64 diagnosed autistic children.

Adjusted for child’s sex and child’s age at completion of the health screen when autism status was first reported.

^a^Including undergraduate or post-graduate degree.

^b^Over the past 12 months, data collected at the 28-week review.

^c^Low socioeconomic index for residential area represent most disadvantage group.

^d^Birth order of the index child among all-live older siblings.

^e^Parent who does not have a partner or parent who has a partner who does not live with them.

^f^Edinburgh Postnatal Depression Scale (EPDS) was administered during at 28-week clinical review.

^g^Maternal history of psychosocial conditions (anxiety, depression, bipolar or other), was collected from antenatal medical records.

^h^Includes selective serotonin reuptake inhibitor, serotonin and norepinephrine reuptake inhibitors and tricyclic antidepressant during pregnancy.

^i^Prenatal adversity scores as previously published by Wang et al (2023) which include information on hypertension, preeclampsia, gestational diabetes, high EPDS score ( ≥13), and maternal pre-pregnancy weight, which was further categorised as obese (BMI ≥ 28) or overweight (24 ≤BMI <28).

^j^Resuscitation at birth involving intubation or cardiac compressions.

Note: For extended list of all factors examined in this scoping study see Table S4.

An additional analysis was conducted, restricting the sample to males, with no changes in the findings. Due to the low number of females, a similar restriction for females was not possible.

*AOR* adjusted odds ratio, *SEIFA* IRSD Socio-Economic Indexes for Areas, Index of Relative Socio-economic Disadvantage, *BMI* body mass index, *NO2* nitrogen dioxide, *PM2.5* particulate matter <2.5 μm, *Ppb* parts per billion, *SEIFA* socioeconomic indexes for areas, *SSRI* selective serotonin reuptake inhibitor, *SNRI* serotonin and norepinephrine reuptake inhibitors, *T1* 1^st^ trimester, *T2* 2^nd^ trimester, *T3* 3^rd^ trimester.

#### Maternal health

The average maternal pre-pregnancy body mass index (BMI) was 25.4 (kg/m²) [SD = 5.5] (Table 1). Half of mothers (*n* = 428) reported any alcohol intake during pregnancy and 7% (*n* = 60) reported any smoking during pregnancy (Table 1). 4.4% (*n* = 27) of mothers scored high ( ≥13) on the Edinburgh Postnatal Depression Scale (EPDS) scale. 9.7% (*n* = 83) reported pre-existing psychosocial conditions, as extracted from antenatal medical records. Overall, 4.2% (*n* = 35) of mothers were taking SSRIs/SNRIs during pregnancy with 3.8% (*n* = 32) using SSRIs/SNRIs in 1^st^ and 2^nd^ trimesters and 2.8% (*n* = 24) using SSRIs/SNRIs in 3^rd^ trimester. For dietary measures, the average Australian Recommended Food Score (ARFS) score was 35.4 [SD = 7.78] out of a maximum possible score of 74 (Table 2).

**Table 2:** Prenatal nutrition, dietary patterns and child autism diagnosis.

Prenatal Nutrition and dietary factors (In increasing daily amounts)	Child’s autism diagnosis^a^			
	Mean [SD]	AOR	95% CI	*P*-value
**Nutrition**				
Folate intake per 100 µg/day	2.71 [0.93]	**0.69**	**0.49,0.95**	**0.025**
Betaine intake per 100 mg/day	4.49 [1.58]	0.87	0.73,1.03	0.107
Total choline per 100 mg/day	3.74 [1.03]	**0.72**	**0.54,0.95**	**0.019**
Free choline per 100 mg/day	1.03 [0.28]	0.45	0.17,1.2	0.112
Phosphatidylcholine per 100 mg/day	1.76 [0.59]	**0.54**	**0.33,0.89**	**0.016**
Phosphocholine per 100 mg/day	0.15 [0.05]	0.01	0,3.49	0.126
Glycerophosphocholine per 100 mg/day	0.60 [0.22]	0.38	0.1,1.44	0.155
Sphingomyelin intake per 100 mg/day	0.19 [0.07]	**0.02**	**0,0.95**	**0.047**
Energy intake 100 kJ/day	74.78 [23.14]	**0.99**	**0.97,1**	**0.048**
All fat per 100 g/day	0.75 [0.26]	0.37	0.12,1.11	0.075
Saturated fat per 100 g/day	0.32 [0.12]	0.14	0.01,1.49	0.104
Polysaturated fat per 100 g/day	0.10 [0.05]	0.02	0,9.01	0.214
Monosaturated fat per 100 g/day	0.26 [0.10]	0.07	0,1.38	0.08
Protein per 100 g/day	0.86 [0.27]	0.35	0.12,1.06	0.064
Carbohydrate per 100 g/day	1.95 [0.62]	0.63	0.39,1.02	0.06
Fibre per 100 g/day	0.22 [0.07]	0.03	0,1.43	0.075
Betacarotene per 100 µg/day	27.42 [12.39]	0.99	0.97,1.02	0.661
Cholesterol per 100 mg/day	2.77 [1.12]	0.80	0.62,1.04	0.091
Magnesium per 100 mg/day	2.91 [0.90]	**0.70**	**0.5,0.97**	**0.032**
Niacin (Vitamin B3) per 100 mg/day	0.20 [0.07]	**0.01**	**0,0.66**	**0.031**
Retinol (Vitamin A1) per 100 µg/day	3.79 [1.52]	0.93	0.78,1.11	0.414
Vitamin C per 100 mg/day	1.27 [0.63]	0.96	0.63,1.46	0.838
Calcium per 100 mg/day	10.25 [3.21]	0.94	0.87,1.02	0.163
Phosphorus per 100 mg/day	15.5 [4.69]	**0.93**	**0.88,0.99**	**0.031**
Potassium per 100 mg/day	28.34 [7.98]	0.97	0.94,1.01	0.104
Sodium per 100 mg/day	23.17 [7.73]	0.96	0.93,1	0.064
Riboflavin (vitamin B2) per mg/day	2.5 [0.9]	**0.68**	**0.49,0.96**	**0.029**
Thiamine (Vitamin B1) per mg/day	1.5 [0.6]	**0.46**	**0.26,0.81**	**0.007**
Omega 3 per g/day	1.4 [0.6]	0.72	0.44,1.19	0.203
Vitamin E per mg/day	6.46 [2.43]	0.89	0.79,1.01	0.071
Iron per mg/day	12.41 [4.39]	**0.9**	**0.84,0.97**	**0.006**
Zinc per mg/day	11.12 [3.6]	**0.91**	**0.84,0.99**	**0.033**
Linoleic acid per 10 mg/day	1.48 [2.12]	**0.86**	**0.74,1**	**0.045**
Lauric acid g/day	1.96 [1.03]	0.75	0.55,1	0.053
**Dietary patterns**				
Glycaemic load (per unit)	98.40 [33.8]	0.99	0.98, 1	0.075
Glycaemic index (per unit)	50.327 [3.49]	1	0.93, 1.08	0.929
Dietary inflammatory index (per unit)	3.493 [1.91]	1.06	0.92, 1.22	0.439
Whole-Foods healthy diet (PC1 z-score per 1 SD)	0.075 [0.99]	0.81	0.61, 1.08	0.146
Processed diet (PC2 z-score per 1 SD)	−0.010 [0.99]	0.86	0.64, 1.15	0.293
Australian Recommended Food Score (per score)	35.405 [7.8]	**0.96**	**0.93, 0.99**	**0.009**

^a^Total cohort *N* = 856 which includes *N* = 64 diagnosed autistic children.

**Bold** indicates an association with a *p* <0.05.

#### Environmental factors and pollutants

The average ambient air pollution at birth for Nitrogen Dioxide (NO_2_) was 5.5 Parts per billion (Ppb) [SD = 2.0] and for Particulate Matter <2.5μm (PM2.5) 7.7 µg/m^3^ [SD = 0.9] (Table 1). 10.6% (*n* = 89) of mothers reported ‘yes’ to exposure to passive tobacco smoke during the three months prior to conception or during the 1^st^ and 2^nd^ trimesters of pregnancy. For housing, 95.0% (*n* = 519) of the cohort lived in a house and 5% (*n* = 27) lived in an apartment, flat or unit. For additional information on household environment refer to Table S3.

#### Birth factors

The average gestational age at birth was 39.4 weeks [SD = 1.5] with 4.3% of births being preterm (32–37 weeks’ gestation vs rest) (Table 1). The average Apgar score at five minutes was 9 [SD = 0.8]. The median duration of labour was 5 h and 0.8% (*n* = 7) of infants required resuscitation at birth.

### Prenatal and birth factors associated with autism diagnosis

#### Demographic and household composition

Several demographic and household composition factors were associated with child’s autism diagnosis by age 11.5 years (Table 1). Male children had higher odds of having autism compared to females (OR = 2.01; 95%CI = 1.17,3.4; *P* = 0.011). Infants of women with a university degree had 41% lower odds of being diagnosed with autism than infants without tertiary education (OR = 0.59; 95% CI = 0.35, 0.98; *P* = 0.043). Being a second or later-born child was associated with 58% lower odds of autism compared to first-born children (OR = 0.42; 95% CI = 0.22, 0.81; *P* = 0.01). Other factors such as paternal education, grandparents’ ancestry and maternal and paternal age were not associated with the child’s odds of autism (Table 1).

#### Maternal health

A higher maternal pre-pregnancy BMI was associated with increased odds of subsequent diagnosis of autism (OR = 1.06 per kg/m² increase; 95%CI = 1.02,1.11; *P* = 0.005). Any alcohol intake during the 3^rd^ trimester was negatively associated (OR = 0.37; 95%CI = 0.17,0.81; *P* = 0.012) with offspring autism (Table S4). Any recreational marijuana use during pregnancy was associated with an increase in child’s autism (OR = 5.68; 95%CI = 1.46,22.62; *P* = 0.014). Maternal pre-existing psychosocial conditions were associated with increased odds of child’s autism (OR = 3.93; 95%CI = 2.10,7.39; *P* < 0.001). SSRI/SNRI use during 1^st^ and 2^nd^ trimester (OR = 6.43 95%CI = 2.76, 14.98; *P* < 0.001) and SSRI/SNRI use during 3^rd^ trimester (OR = 9.01 95%CI = 3.70,21.9; *P* <0.01) was also associated with increased odds of the child having autism. Other maternal health measures such as preeclampsia, hypertension and gestational diabetes mellitus were not separately associated with autism. However, greater prenatal adversity (a combination of preeclampsia, hypertension, gestational diabetes, obesity and an EPDS score ≥13^36^) was associated with higher odds of autism (one of the nominated adversities vs none: OR = 2.04; 95%CI = 1.09,3.82; *P* = 0.025; two or more adversities vs none: OR = 2.36; 95%CI = 1.26,4.42; *P* = 0.008).

#### Maternal nutrition during pregnancy

Various indices of prenatal nutrition were associated with a reduced likelihood of subsequent autism in the offspring (Table 2). These included folate (OR = 0.69 per 100 µg/day increase; 95%CI = 0.49,0.92; *P* = 0.025), total choline (OR = 0.72 per 100 mg/day increase; 95%CI = 0.54,0.95; *P* = 0.019), magnesium (OR = 0.7 per 100 mg/day increase; 95%CI = 0.5,0.97; *P* = 0.032), niacin (OR = 0.01 per 100 mg/day increase; 95%CI = 0,0.66; *P* = 0.031), riboflavin (vitamin B2) (OR = 0.68, per g/day increase; 95%CI = 0.49,0.96; *P* = 0.029), thiamine (vitamin B1) (OR = 0.46 per g/day increase; 95%CI = 0.26,0.81; *P* = 0.007), iron (OR = 0.9 per mg/day increase; 95%CI = 0.84,0.97; *P* = 0.006), zinc (OR = 0.91 per mg/day increase; 95%CI = 0.84,0.99; *P* = 0.033), and phosphorus (OR = 0.93 per mg/day increase; 95%CI = 0.88,0.99; *P* = 0.031).

Overall dietary patterns were also examined; higher adherence to the Australian Dietary Guidelines represented by the ARFS was associated with lower odds (OR = 0.96; 95%CI = 0.93,0.99; *P* = 0.009) for autism in offspring (Table 2).

Additionally, supplementation during pregnancy was also examined (Table S5, Table S6). In a sensitivity analysis, we also adjusted for maternal energy intake (per kJ/day) (Table S7).

#### Environmental factors and pollutants

Maternal exposure to passive tobacco smoke 3 months prior to conception and/or during the 1^st^ and/or 2^nd^ trimesters was associated with higher odds of their child having autism (OR = 2.64; 95%CI = 1.38,5.05; *P* = 0.003) (Table 1). Vinyl flooring in the living area during pregnancy was associated with increased odds of autism (OR = 4.21; 95%CI = 1.45,12.21; *P* = 0.008). The estimated effect of gas heater use during pregnancy on the odds of autism was OR = 1.94; 95%CI = 0.95,3.94; *P* = 0.067. Exposure to other markers of environmental pollutants, such as pesticide use, ambient air pollution at birth and signs of mould/dampness, was not found to associate with autism (Table 1).

#### Birth factors

Preterm birth (32–37 weeks vs rest) (OR = 2.58; 95%CI = 1.02,6.51; *P* = 0.044) was associated with increased odds of child autism (Table 1). Other birth factors such as placental weight, gestational age at birth, Apgar score at 5 min, labour duration (hours) and delivery mode were not found to be associated with child’s autism (Table 1, Table S4).

### Correlation of prenatal and birth factors

**Fig. 2:**
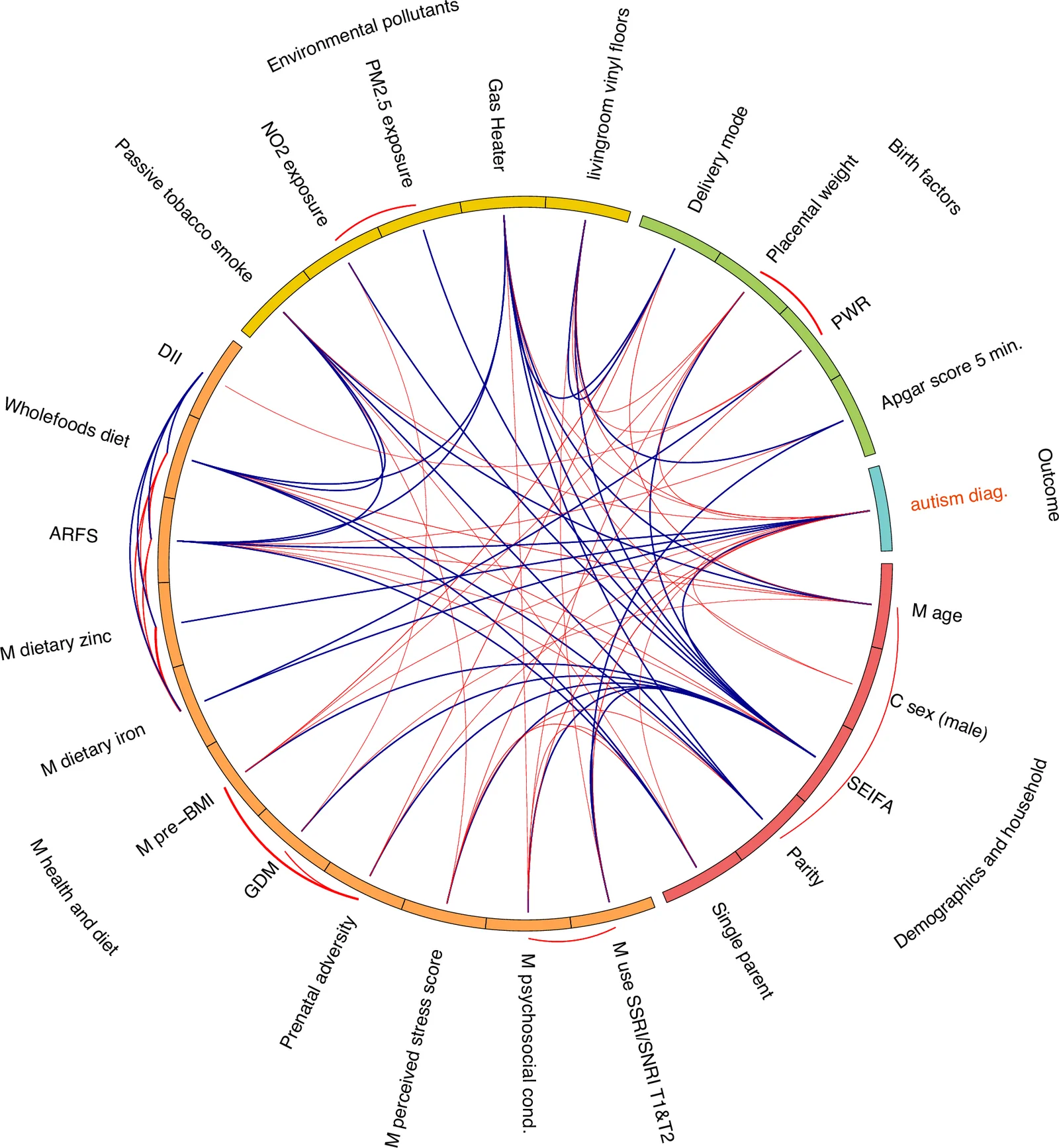
Correlogram of prenatal and birth factors and child autism diagnosis. A correlogram of demographic and household composition, maternal health and diet, maternal environmental pollutants and birth factors and autism diagnosis. Inter-domain correlations with >-0.23 and >0.23 and intra-domain correlations with magnitude >-0.07 and >0.08 respectively are displayed. Red lines represent positive correlations, and blue lines represent negative correlations. Pearson’s correlation was used for pairs of continuous and ordinal variables; point-biserial correlation was used for correlation between continuous and binary variables; phi coefficient was used for co1rrelation between two binary variables. DII Dietary inflammatory index, ARFS Australian recommended food score, BMI Body mass index, GDM gestational diabetes mellitus, SSRI Selective serotonin reuptake inhibitors, SNRI Serotonin and norepinephrine reuptake inhibitors, PWR Placenta-to-birth weight ratio, M maternal, C child, SEIFA Socioeconomic Indexes For Area, PA Prenatal adversity.

Prenatal and birth factors across various domains (maternal health and diet, maternal environmental pollutants, demographic and household, birth factors) were highly correlated (Fig. 2, Fig. S1). As an example, the wholefoods diet and the ARFS were positively correlated (R = 0.56, *P* <0.001). This highlights the co-occurrence patterns among many environmental factors.

### Qualitative integration of current findings with existing literature on prenatal and birth factors

Many of the findings that we report above have prior evidence of association with autism, thereby strengthening their relevance. For example, there is evidence from large reviews that maternal BMI, maternal alcohol use, maternal smoking and outdoor air pollution during pregnancy are associated with childhood neurodevelopmental delays and autism^28,29,37,38^ (Table S1).

**Fig. 3:**
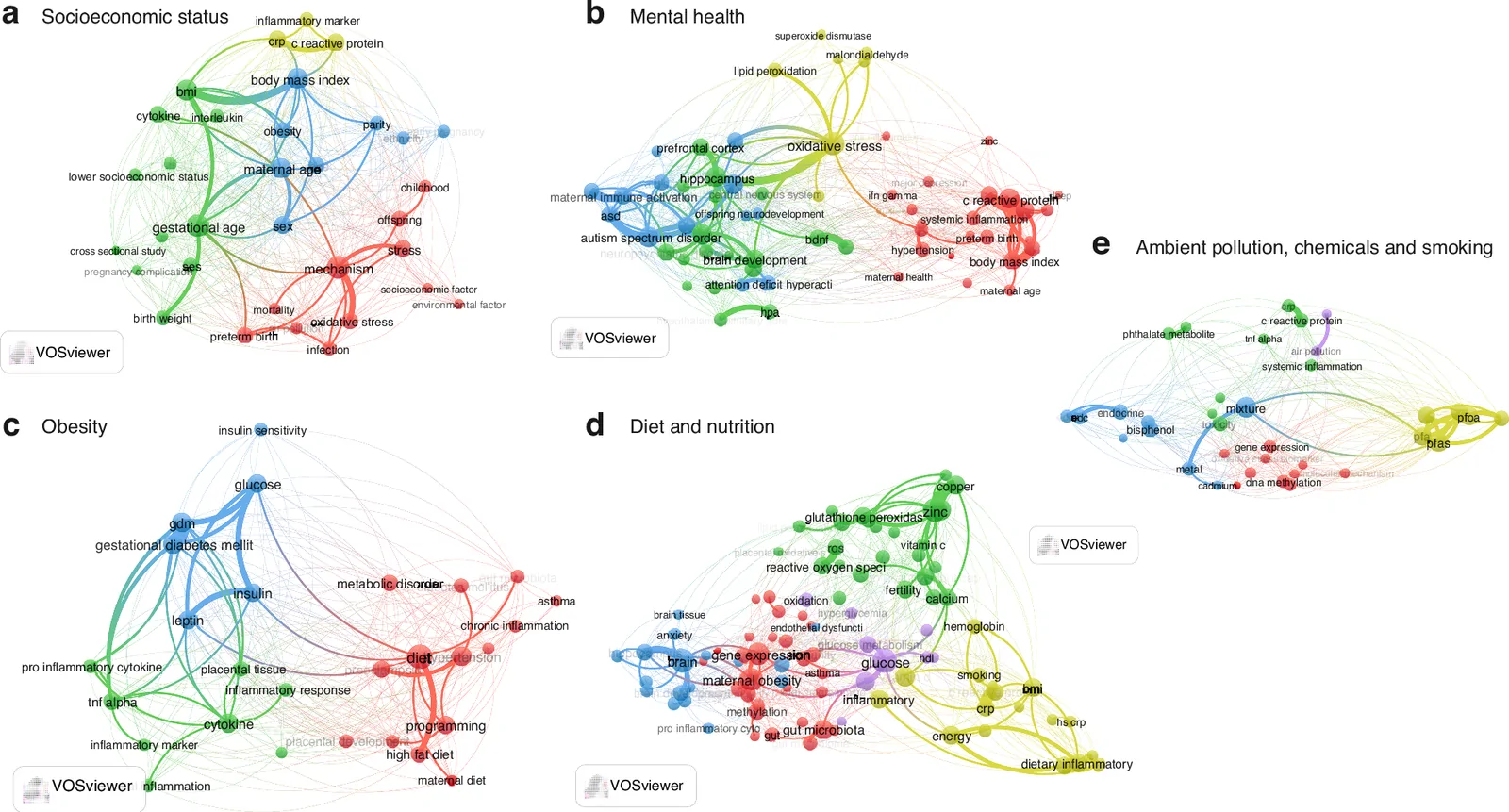
VOSviewer literature network analysis linking prenatal environmental exposures with inflammation and oxidative stress. Panels represent clusters on (**a**) socioeconomic status, (**b**) mental health, (**c**) obesity, (**d**) diet and nutrients, and (**e**) ambient pollution, chemicals, and smoking.

Interrelated risk factors may act through shared pathways. To support this, we draw on existing literature and emphasise the need for future research to examine these pathways when investigating the causality of such multifaceted relationships. Table S2 summarises current literature evidence on common mechanisms of action, particularly oxidative stress and inflammation, through which these exposures may act. Additionally, Fig. 3 outlines the literature network overlap of oxidative stress and inflammation in key exposures such as socioeconomic status, maternal mental health, obesity, diet and nutrition and environmental pollutants. Socioeconomic status, maternal mental health and diet had the most literature related to oxidative stress and inflammation.

## Discussion

We examined prospective associations between a wide range of prenatal and birth factors and the likelihood of the child receiving an autism diagnosis by a paediatrician against DSM-5 criteria, with the goal of identifying possible early life factors acting through unifying pathways to increase the likelihood of autism. This approach revealed important patterns that will inform future investigations. Lower socioeconomic position correlated with many factors as well as with increased likelihood of autism. Pre-existing maternal mental health conditions and SSRI/SNRIs use during pregnancy were associated with a substantially increased risk of autism. There are a variety of potential causal and non-causal explanations for this finding, which, given the public health significance, clearly warrant further investigation. Passive tobacco smoke before conception and 1^st^ and 2^nd^ trimesters and exposure to vinyl floors in the living room during pregnancy were positively associated with the infant subsequently being diagnosed with autism. Conversely, many prenatal nutritional indices were inversely associated with autism. Furthermore, higher adherence to the Australian Dietary Guidelines, as indicated by higher ARFS, was associated with a reduced risk of child autism. These results are consistent with evidence that pregnancy is a critical period for early life programming that can have lifelong effects.^39^

### Maternal health

Maternal physical and mental health has been associated with an increased likelihood of autism in children. Conditions such as obesity, gestational diabetes, and preeclampsia have been shown to impact foetal brain development,^40–42^ as well as being associated with increased likelihood of autism.^43,44^ In our study there was no evidence that preeclampsia, hypertension and gestational diabetes mellitus were individually associated with autism, possibly due to low power. However, exploring combined factors such as a previously established metric of ‘prenatal adversity’, which includes each of these pregnancy complications and has been linked to inflammation,^36^ was associated with a greater likelihood of the child being diagnosed with autism. Our approach is consistent with the argument that combination effects or prenatal comorbid exposures may act synergistically to exacerbate the risk of neurodevelopmental conditions.^45^

Maternal mental health has previously been associated with offspring autism. Lu et al. (2022) found that depression during pregnancy was associated with an 75% increase in the risk for autism diagnosis in the child.^46^ Similarly, the Environmental Influences on Child Health Outcomes program combined 33 prenatal/paediatric cohorts (*n* = 3994) and reported prenatal depression was associated with an increase in moderate to severe autism-related traits by 64%.^47^ Further to this, the use of SSRIs during pregnancy has been associated with an increased likelihood of offspring autism in the literature.^48,49^ A large record linkage study reported that such an association did not persist after adjustment for factors such as socioeconomic factors, psychiatric history before birth and perinatal factor—gestational age.^50^ In our setting, Pham et al. reported associations between SSRI use during pregnancy and perinatal factors such as low Apgar scores and need for resuscitation at birth. Perinatal factors may represent downstream consequences of exposure; therefore, routine adjustment should not be made without considering these issues.^51^ Maternal SSRI use may be a consequence of underlying maternal psychosocial conditions.^18,52^ In addition inflammation during pregnancy may play an antecedent role in the development of both (i) psychiatric conditions such as depression that are a trigger to SSRIs use^53,54^ as well as (ii) offspring autism.^55^ Alternatively, maternal SSRI/SNRIs use during pregnancy may be causally linked to child autism via pathways such as oxidative stress.^55^ Therefore, careful evaluation of causality via cohort studies with investigation of mediation pathways and accompanying experimental work, is a priority.

### Maternal nutrition and supplementation

Prenatal nutrition is emerging as a key determinant of offspring neurodevelopment.^56^ Multiple prenatal dietary micronutrients and minerals have been shown to be associated with reduced likelihood of a child’s autism including folate, choline, zinc, and iron.^57–60^ Studies from the Childhood Autism Risks from Genetics and Environment case (CHARGE) and In the Markers of Autism Risk in Babies: Learning Early Signs (MARBLES) cohorts found that prenatal vitamin use during early pregnancy was associated with a significantly reduced risk (40–50%) of having an autistic child.^61,62^ However, it is important to note that many prenatal multivitamins differ in dosage and nutrient composition, leading to measurement misclassification, which may contribute to null results reported in some studies.^63^

From a dietary pattern perspective, eating in alignment with the Australian Dietary Guidelines during pregnancy reduced likelihood of child autism, suggesting that food variety and nutrient density are important during pregnancy. A recent study examining dietary patterns reported a significant reduction in the likelihood of child’s autism across two large cohorts, after high adherence to prenatal healthy dietary pattern.^64^ Therefore, the prenatal dietary patterns and their association with an increased likelihood of autism needs further evaluation.

Moreover, a review has been conducted examining diet as a potential modifying factor of other environmental factors in autism.^26^ In fact, 10 out of the 12 studies identified prenatal dietary factors as a significant modifier attenuating the associations between adverse environmental exposure and autism. This review emphasised the importance of understanding the mechanisms of combined environmental exposures on neurodevelopmental outcomes.^26^

### Prenatal pollution and environmental chemicals

Prenatal environmental pollutants have been reported to increase likelihood of autism in the offspring. Previously, studies have linked the mother’s urinary metabolome, including nicotine metabolites and exposure to second-hand tobacco smoke, with increased autism in the offspring.^65,66^ A study, including a meta-analysis of over 200,000 participants, reported that marijuana use during pregnancy increased the likelihood of autism in offspring.^67–69^ Ambient air pollution has also been linked to autism^70,71^ as shown previously to be associated with autism symptoms at age two in this study.^17^ We found that vinyl flooring was associated with a child’s autism diagnosis in part due to exposure to pollution in the home. Previous studies have reported a similar association for vinyl flooring implicating chemicals such as phthalates.^72,73^ These chemicals are neurotoxic and are associated with an increased likelihood of autism^74^; however, further research is needed to strengthen the link between the neurotoxic chemicals and the underlying mechanisms in autism.

Pollutant exposure measures derived from biosamples, such as maternal exposure to the endocrine-disrupting plastic-associated chemicals bisphenols and phthalates have been previously studied in the BIS cohort Childhood Autism Risks from Genetics and Environment case (CHARGE)^22,75^ and were outside of the scope of this current study.

### Socioeconomic disadvantage

Our findings complement and extend previous research linking greater socioeconomic disadvantage with autism diagnosis in the offspring.^76–78^ Previous research from different countries, including Japan, Taiwan, Denmark and Sweden, found that low socioeconomic disadvantage is associated with a higher likelihood of autism, as we observed,^77,79,80^ whereas other studies have not.^78,81,82^ Socioeconomically disadvantaged groups are at a heightened likelihood of autism, potentially due to a range of contributing factors. They often face greater challenges during pregnancy, including complications such as preeclampsia and gestational diabetes.^83^ Additionally, higher rates of smoking, obesity, and poor dietary habits are prevalent among these groups, which can further exacerbate this risk.^84^ We reported in 2024 that, in BIS, maternal diet mediated the association between socioeconomic adversity and poorer offspring cognition by 22% and suggested that modifiable maternal diet may partially explain the association between socioeconomic adversity and child neurocognitive vulnerability.^85^ From a public health perspective, addressing modifiable factors such as diet, pollutant exposure, and obesity – if causal - should be integral to the development and implementation of public health policies.

### Strengths and weaknesses

The strengths of this study include the unselected antenatal sampling frame, the high retention rate (84% to the 2020–2022 child health screen), and that we were able to review the medical records of 79 of the 80 (99%) children with parent-reported autism to identify the 64 children with a diagnosis of autism made by a paediatrician against the DSM 5 criteria. Further, we have assembled and examined a uniquely comprehensive set of environmental factors, on the premise that multiple factors will likely operate via unifying biological pathways. We have used both prior knowledge and data-driven approaches to identify and examine over 100 early life factors relevant to changes in the likelihood of autism in the offspring. Many factors correlated, consistent with multiple environmental factors operating via unifying biological pathways to autism development (Fig. 2, Fig. S1). We did not correct for multiple hypothesis testing because these exposures are unlikely acting independently and could increase the type II error in this exploratory study.^7^ Additionally, due to the nature of this approach including wide range of factors examined, fully adjusted multivariate analysis was not performed including potential confounding factors. The results here warrant future consideration including the exploration of shard common pathways across several exposures of interest, and of course, replication. In BIS, considering multiple exposures simultaneously has highlighted their convergence on shared biological pathways. For example, Ponsonby et al. showed that prenatal exposure to plastics, maternal smoking during pregnancy and no fish oil supplementation have been linked to altered oxidative stress which is further linked to autism symptoms in children.^22^ Additionally, Tanner et al. applied environmental mixtures analysis incorporating several of the potential autism risk factors identified in this study, and reported an overall mixture association with epigenetic changes in a key gene previously linked with autism in males.^21,31^ We acknowledge possible misclassification when deriving daily dietary nutrient measures from the food frequency questionnaire and environmental pollutants such as pesticide use during pregnancy, particularly as most of these measures are self-reported. While we have not examined confounders relevant to health service presentation and delivery, standardised criteria were applied across clinical services. Gene-environment interactions modulate neurodevelopment, therefore, these prenatal and birth factors may interact differently with an individual’s genotype.^22^ Finally, we look at autism as a single neurodevelopmental phenotype and in isolation of potentially co-existing neurodevelopmental factors. Further studies are required that take into consideration co-existing neurodevelopmental diagnoses such as Attention-Deficit/Hyperactivity Disorder. Finally, the BIS cohort is relatively homogeneous with respect to education level, socioeconomic status, and ethnicity. As a result, further work needs done to generalise our findings to other populations.

### Future directions on proposed mechanisms of interest

Our findings reinforce that environmental factors associated with autism are multifactorial. Many of these environmental features or exposures may act by common pathways to promote pro-inflammatory and pro-oxidative profiles.^22,53^ We examined some of the features in this study in the context of inflammation and oxidative stress in literature, which is depicted in Table S1. These processes also impact on mitochondria dysfunction which is increasingly recognised to be an autism feature.^12,13^ This scoping study was designed to identify possible early life factors altering subsequent autism risk. Future work could be conducted using several approaches. Firstly, exposures that have been identified as potential autism risk factors and have established linked to the same biological pathway, such as inflammation, could be combined into composite scores. For example, we have previously reported that the following are associated with prenatal inflammation: maternal obesity, higher parity, pre-existing psychosocial conditions and passive smoking in pregnancy.^53,85^ Thus, these factors should be considered as possible co-exposures in an inflammatory pathway. Secondly, future research should employ multivariable analyses integrating directed acyclic graphs to identify and adjust for confounders, enabling in-depth examination of key exposures and shared biological pathways.^27^ For example, for SSRI use during pregnancy, one would need to evaluate the indication for SSRI use (such as depression), replicate these results in an external cohort and understand the putative mechanistic impacts, including epigenetics, lipidomics and mitochondrial pathways to fully understand the causal relationships. In our future work, we will study composite exposure associated with autism and use multivariable models to test biological mediation, as well as considering genetic predisposition, as outlined in our work regarding modern approaches to causal inference in highly dimensioned studies.^24,85,86^

In conclusion, our findings highlight that the prenatal period is critical for early life programming of autism. A diverse range of prenatal and birth factors, many of which are modifiable, may contribute to the aetiology of autism through shared mechanistic pathways. If multiple modifiable environmental factors influence child development through shared mechanistic pathways, future research could improve our understanding of neurodevelopmental outcomes by examining the combined effects of these factors. The focus should be on identifying unifying, targetable mechanisms and developing composite exposure measures, rather than studying single exposures in isolation.

## Supplementary information

Revised_formatting_Supplement tables_longer_than_one_page_TableS1-S4_R1_09_10_2025

TableS5_07_10_2025

TableS6_07_10_2025

TableS7_07_10_2025

Supplementary_methods_9_10_2025

Supplementary_results_07_10_2025

Figure_S1_07_10_2025

Box_S1_07_10_2025

BoxS2_07_10_2025

## Data Availability

Access to BIS data including all data used in this paper can be requested through the BIS Steering Committee by contacting the corresponding author. Requests to access cohort data are considered on scientific and ethical grounds and, if approved, provided under collaborative research agreements. Deidentified cohort data can be provided in Stata or CSV format. Additional project information, including cohort data description and access procedures, is available at the cohort study’s website http://www.barwoninfantstudy.org.au.
