# Correction: Prenatal and birth factors associated with child autism diagnosis: a birth cohort perspective

**DOI:** 10.1038/s41390-026-04831-w

**Published:** 2026-02-25

**Authors:** Lada Holland, Katherine Drummond, Sarah Thomson, Luba Sominsky, Wolfgang Marx, Chloe Love, Samantha L. Dawson, Leonard C. Harrison, Richard Saffery, Christos Symeonides, Mimi LK Tang, David Burgner, Peter D. Sly, Peter Vuillermin, Anne-Louise Ponsonby, the BIS Investigator group, Toby Mansell, Sarath Ranganathan, Martin O’Hely

**Affiliations:** 1 The Florey Institute of Neuroscience and Mental Health, The University of Melbourne, Parkville, VIC Australia; 2 Child Health Research Unit, Barwon Health, Geelong, VIC Australia; 3 Institute of Mental and Physical Health and Clinical Translation, School of Medicine, Deakin University, Geelong, VIC Australia; 4 Murdoch Children’s Research Institute, Parkville, VIC Australia; 5 Walter and Eliza Hall Institute of Medical Research, Melbourne, VIC Australia; 6 Centre of Community Child Health, Royal Children’s Hospital, Parkville, VIC Australia; 7 Minderoo Foundation, Perth, Australia; 8 Department of Paediatrics, University of Melbourne, Parkville, VIC Australia; 9 Children’s Health and Environment Program, Child Health Research Centre, The University of Queensland, South Brisbane, QLD Australia

Correction to: *Pediatric Research* 10.1038/s41390-025-04747-x;published online 15 January 2026

In this article the BIS Investigator group members Toby Mansell, Sarath Ranganathan and Martin O’Hely have been removed from the general author list and added to the consortium. The original article has been corrected.

