# Supplementary material for: Prenatal and birth factors associated with child autism diagnosis: a birth cohort perspective
Source: Pediatr Res. 2026 Jan 15;100(3):1129–42. doi: 10.1038/s41390-025-04747-x (PMC13672113; doi:10.1038/s41390-025-04747-x)
Supplement: Supplementary file 1 [file 41390_2025_4747_MOESM2_ESM.pdf]

Table S5 Prenatal daily supplementation and child autism diagnosis

Supplementation during pregnancy	Child's autism diagnosis[1]			
	% (N) or Mean [SD](N)	AOR	95% CI	P-value
Folic acid supplementation				
lowest quintile (0 g/day)	28.4% (237)		Reference	
rest (0.2-2 g/day)	71.6% (597)	0.61	0.35,1.07	0.083
Folate and folic acid ingested				
lowest quintile (0 .078-0.365g/day)	25.6% (204)		Reference	
rest (0.376-2.259 g/day)	74.4% (594)	**0.54**	**0.30,0.96**	**0.036**
Iron supplementation (mg/day)[a]				
lowers quintile (0 mg/day)	35.4% (295)		Reference	
rest (5-165 mg/day)	64.6% (539)	0.91	0.52,1.58	0.737
Iron total ingested (mg/day)				
lowest quintile (3.4-12.97 mg/day)	23.8% (190)		Reference	
rest (13.22-179.1 mg/day)	76.2% (608)	0.84	0.45,1.56	0.580
Choline supplementation >50mg/day : yes[a]	2.6% (22)	1.3	0.29,5.77	0.732
Choline supplement ingested (100 mg/day)	3.8 [1.1] (800)	0.81	0.61,1.08	0.147
Zinc supplementation[a]				
lowest quintile (0 mg/day)	32% (267)		Reference	
rest (1.3-46 mg/day)	68% (567)	**0.59**	**0.34,1.01**	**0.06**
Zinc total ingested (10mg/day)	2.0 [0.9] (798)	**0.71**	**0.51,0.99**	**0.047**
Magnesium supplementation[a]				
lowest quintile (0 mg/day)	36% (300)		Reference	
rest (10-752 mg/day)	64% (534)	0.59	0.34,1.01	0.055
Magnesium total ingested (100 mg/day)	3.4 [1.1] (798)	**0.72**	**0.54,0.96**	**0.028**
Fish oil supplementation: yes	54.5% (452)	1.11	0.64,1.91	0.719
Vitamin D supplementation: yes	91.0% (754)	1.39	0.49,3.98	0.538

[1]Total cohort N=856 which includes N=64 diagnosed autistic children

Bold indicates an association with a $p < 0.05$

Adjusted for child's sex and child's age at completion of the health screen when autism status was first reported.

[a]Due to the nature of supplementation, the lack of normal distribution did not allow for examination on a continuous scale.
