# Supplementary material for: Prenatal and birth factors associated with child autism diagnosis: a birth cohort perspective
Source: Pediatr Res. 2026 Jan 15;100(3):1129–42. doi: 10.1038/s41390-025-04747-x (PMC13672113; doi:10.1038/s41390-025-04747-x)
Supplement: Supplementary file 2 [file 41390_2025_4747_MOESM3_ESM.pdf]

Table S6 Dietary folate and folic acid supplementation, including recommended intake and its association with child's autism diagnosis

Dietary folate and folic acid supplementation	Autism diagnosis			
	% (N)	AOR	95% CI	P-value
Dietary folate intake				
Q1 (0.07-0.21 1000µg/day)	25% (205)		Reference	
Q 2 (0.21-0.26 1000µg/day)	25% (205)	0.62	0.31,1.24	0.179
Q 3 (0.26-0.32 1000µg/day	25% (205)	0.60	0.30,1.22	0.157
Q 4 (0.32-0.82 1000µg/day)	25% (205)	*0.48*	0.22,1.02	0.056
Folic acid supplementation				
Q1 (0 1000µg/day)	28% (232)		Reference	
Q2 (0.03-0.4 1000µg/day)	23% (190)	**0.36**	**0.15,0.87**	**0.023**
Q3 (0.45-0.8 1000µg/day)	39% (324)	0.72	0.38,1.35	0.304
Q4 (0.85-2 1000µg/day	10% (88)	0.80	0.32,1.99	0.643
Folate and folic acid ingested				
Q1 (0.07-0.37 1000µg/day)	25% (200)		Reference	
Q2 (0.37-0.73 1000µg/day)	25% (199)	**0.34**	**0.15,0.80**	**0.013**
Q3 (0.73-1.03 1000µg/day)	25% (200)	0.69	0.34,1.41	0.312
Q4 (1.03-2.26 1000µg/day)	25% (199)	0.57	0.27,1.20	0.138
Recommended intake of ingested folate and folic acid[a]				
Below recommended intake	35% (304)		Reference	
Meeting recommended intake	30% (256)	0.83	0.43,1.60	0.583
Exceeding recommended intake	35% (296)	0.96	0.52,1.76	0.898

Bold indicates an association with a $p<0.05$

[a]Recommended intake as per the Nutrient Reference Values for Australia and New Zealand, including recommended dietary intakes published in 2006[19]

Adjusted for child's sex and child's age at completion of the health screen when autism status was first reported.
