# Supplementary material for: Prenatal and birth factors associated with child autism diagnosis: a birth cohort perspective
Source: Pediatr Res. 2026 Jan 15;100(3):1129–42. doi: 10.1038/s41390-025-04747-x (PMC13672113; doi:10.1038/s41390-025-04747-x)
Supplement: Supplementary file 3 [file 41390_2025_4747_MOESM4_ESM.pdf]

Table S7 The associations between prenatal nutrition, dietary factors, and autism diagnosis

Nutrition and dietary factors	Child's autism diagnosis[1]		
	AOR	95% CI	P-value
**Nutrition**			
Folate intake per 100µg/day	0.75	0.46,1.21	0.236
Betaine intake per 100mg/day	0.92	0.76,1.12	0.401
Total choline per 100mg/day	0.74	0.5,1.1	0.135
Free choline per 100mg/day	0.72	0.2,2.58	0.615
Phosphatidylcholine per 100mg/day	0.59	0.32,1.09	0.092
Phosphocholine per 100mg/day	0.09	0,60.06	0.468
Glycerophosphocholine per 100mg/day	0.69	0.15,3.23	0.633
Sphingomyelin intake per 100mg/day	0.05	0,6.44	0.229
All fat per 100g/day	1.65	0.06,42.6	0.762
Saturated fat per 100g/day	1.73	0.02,177.72	0.816
Polysaturated fat per 100g/day	1.73	0,4107.9	0.89
Monosaturated fat per 100g/day	2.94	0,9487.47	0.793
Protein per 100g/day	0.81	0.07,9.34	0.864
Carbohydrate per 100g/day	0.95	0.25,3.57	0.939
Fibre per 100g/day	0.27	0,99.34	0.664
Betacarotene per 100µg/day	1.01	0.98,1.04	0.455
Cholesterol per 100mg/day	0.93	0.65,1.35	0.711
Magnesium per 100mg/day	0.76	0.41,1.41	0.381
Niacin (Vitamin B3) per 100mg/day	0.02	0,53.32	0.336
Retinol (Vitamin A1) per 100µg/day	1.11	0.87,1.41	0.41
Vitamin C per 100mg/day	1.29	0.79,2.12	0.307
Calcium per 100mg/day	0.99	0.89,1.11	0.891
Potassium per 100mg/day	1.01	0.94,1.08	0.857
Sodium per 100mg/day	0.99	0.91,1.09	0.913
Riboflavin (vitamin B2) per mg/day	0.76	0.47,1.23	0.265
Thiamine (Vitamin B1) per mg/day	0.42	0.17,1.03	*0.058*
Omega 3 per g/day	1.25	0.57,2.75	0.579
Vitamin E per mg/day	0.96	0.79,1.16	0.654
Iron per mg/day	**0.87**	**0.76,0.99**	**0.04**
Zinc per mg/day	0.93	0.78,1.1	0.396
Linoleic acid per 10mg/day	0.87	0.75,1.02	0.079
Lauric acid g/day	0.85	0.54,1.32	0.47
**Dietary patterns**			
Glycemic load	1	0.98,1.02	0.976
Glycemic index	1.02	0.95,1.09	0.663
Dietary inflammatory index	0.93	0.77,1.12	0.415
Whole-Foods healthy diet	0.87	0.64,1.17	0.359
Processed diet	1.39	0.81,2.37	0.231
Australian Recommended Food Score	**0.96**	**0.93,1**	**0.034**

Bold indicates an association with a $p<0.05$

[1] Total cohort N=856 which includes N=64 diagnosed autistic children

Adjusted for child's sex and child's age at completion of the health screen when autism status was first reported and energy intake during pregnancy.

To ensure estimates are more interpretable, some macronutrients and micronutrients were converted to 100unit/day.
