# Supplementary material for: Prenatal and birth factors associated with child autism diagnosis: a birth cohort perspective
Source: Pediatr Res. 2026 Jan 15;100(3):1129–42. doi: 10.1038/s41390-025-04747-x (PMC13672113; doi:10.1038/s41390-025-04747-x)
Supplement: Supplementary file 4 [file 41390_2025_4747_MOESM5_ESM.pdf]

Supplementary methods

Demographic and household derived variables

Household income was recorded as the mean of the values during pregnancy and one year after birth. Index of Relative Socioeconomic Disadvantage (IRSD) was based on the residential address from the Census of Population and Housing: Socioeconomic Indexes For Area (SEIFA) in Australia 2016 [1]. Birth order refers to the index child's birth relative to live-born older siblings.

Maternal health and diet derived variables

Perceived stress score [2], Dietary Inflammatory Index (DII) [3], and Australian Recommended Food Score (ARFS) [4] and Whole-Foods healthy diet and processed diet [3, 5] were included as these factors have been previously demonstrated to be relevant to neurodevelopmental outcomes. Information on antidepressant use, such as selective serotonin reuptake inhibitors (SSRIs), or serotonin and norepinephrine reuptake inhibitors (SNRIs) use was collected in the medication questionnaire during 1st and 2nd trimester. All dietary indices were calculated from the Dietary Questionnaire for Epidemiological Studies Version 2 (DQESv2) [6]. Supplementation during pregnancy was reported as any daily supplementation during pregnancy combined from self-reported questionnaires collected at two timepoints (28 weeks of gestation, 4 weeks after birth). Supplements were taken in the form of either a multivitamin or multimineral formulation, or as separate capsules. Total nutrient ingested was reported by adding both supplementation and dietary intake. Ingested folate and folic acid was also examined in quartiles and also reported as folate intake being below or meeting or above recommended intake of 600µg/day. For active and passive tobacco smoke exposure, we considered any tobacco smoke exposure (yes vs. no) across the 3-month pre-conception period, and the 1st, 2nd and 3rd trimesters. For alcohol intake we included any alcohol intake (yes vs no) during pregnancy, including the 1st, 2nd and 3rd trimesters. The Edinburgh Postnatal Depression Scale (EPDS) questionnaire [7] was administered at 28 weeks of gestation during the in-clinic appointment and the EPDS score was derived as published previously [8]. Maternal height and weight were measured during the in-clinic appointment at 28 weeks of gestation. Pre-pregnancy weight was self-reported during the in-clinic appointment, and a body mass index (BMI) measure was derived. Lastly, a prenatal adversity score was derived as published previously [9]. This score included prenatal conditions such as hypertension, preeclampsia,

gestational diabetes, high EPDS score (≥13), and maternal pre-pregnancy weight, which was further categorised as obese (BMI≥28) or overweight (24 ≤ BMI < 28). Based on the number of prenatal adversities, participants were categorized into three mutually exclusive groups: no PEA, 1 PEA, and ≥ 2 PEA.

Environmental factors and pollutants

Outdoor ambient pollution in pregnancy was previously published in relationship to autism symptoms [10] and lung fuction [11] in Barwon Infant study (BIS). In brief, residential address was used to estimate satellite-based nitrogen dioxide and $PM_{2.5}$ particles at each infant's residential address (2010–2013) [12].

The household data were collected through a questionnaire during the child's year two in-clinic study visit, which included information such as building type, heating, cooling, cooking, mould, dampness (for full list see Box S1) for both the current and previous residence. However, for the purpose of this analysis, we restricted this data to the household the mother reported during pregnancy only as the scope of this analysis focused on prenatal and birth factors. Pesticide use during pregnancy was collected during questionnaire administered at two timepoints (28 weeks of gestation, 4 weeks after birth).

Birth factors

Birth factors, such as gestational age, birth weight, Apgar scores, mode of delivery and resuscitation measures, were collected prospectively from hospital records [13]. Placenta-to-birth weight ratio has been previously published showing association with maternal smoking and alcohol consumption [14].

Sparse bias

Six exposure variables (any recreational marijuana use during pregnancy, single parent, vinyl flooring in the living room, preterm birth (32-37 weeks vs 38 weeks or more), SSRIs/serotonin and norepinephrine reuptake inhibitors (SNRIs) use in 1st and 2nd trimester, SSRIs/SNRIs use in 3rd trimester) had 5 or fewer male and/or 5 or fewer female cases in the exposed category. With few study participants at combinations of the outcome and covariate levels, the maximum likelihood estimate of the odds ratio can be biased away from the null [15]. In a sensitivity analysis, we ran Bayesian penalised logistic regression models using a F (2,2) prior distribution to address potential sparse bias as suggested by Greenland et al. (2016). In a recent simulation study, Bayesian approaches were found to be superior to other methods for addressing sparse bias [16].

Reference:

1. Australian Bureau of Statistics. Socio-Economic Indexes for Areas (SEIFA): Technical Paper 2021 [Available from: https://www.abs.gov.au/statistics/detailed-methodology-information/concepts-sources-methods/socio-economic-indexes-areas-seifa-technical-paper/latest-release.
2. Cohen S, Kamarck T, Mermelstein R. Perceived stress scale. Measuring stress: a guide for health and social scientists. J Health Soc Behav 1994;24:386–96.
3. Gogos A et al. Socioeconomic adversity, maternal nutrition, and the prenatal programming of offspring cognition and language at two years of age through maternal inflammation. Brain Behav Immun. 2024;122:471-82.
4. Collins CE, Young AF, Hodge A. Diet quality is associated with higher nutrient intake and self-rated health in mid-aged women. J Am Coll Nutr. 2008;27(1):146-57.
5. Dawson SL et al. Maternal prenatal gut microbiota composition predicts child behaviour. EBioMedicine. 2021;68:103400.
6. Giles G, Ireland P. Dietary questionnaire for epidemiological studies (version 2). The Cancer Council Victoria, Melbourne,. 1996.
7. Cox JL, Holden JM, Sagovsky R. Detection of postnatal depression. Development of the 10-item Edinburgh Postnatal Depression Scale. Br J Psychiatry. 1987;150:782-6.
8. Mansell T et al. Maternal mental well-being during pregnancy and glucocorticoid receptor gene promoter methylation in the neonate. Dev Psychopathol. 2016;28(4pt2):1421-30.
9. Wang H et al. Prenatal environmental adversity and child neurodevelopmental delay: the role of maternal low-grade systemic inflammation and maternal anti-inflammatory diet. Eur Child Adolesc Psychiatry. 2023.
10. Pham C et al. Early life environmental factors associated with autism spectrum disorder symptoms in children at age 2 years: A birth cohort study. Autism. 2022;26(7):1864-81.
11. Vilcins D et al. Association of maternal air pollution exposure and infant lung function is modified by genetic propensity to oxidative stress. medRxiv. 2023.
12. Knibbs L et al. Independent Validation of National Satellite-Based Land-Use Regression Models for Nitrogen Dioxide Using Passive Samplers. Environmental Science & Technology. 2016;50(22):12331-8.
13. Vuillermin P et al. Cohort Profile: The Barwon Infant Study. Int J Epidemiol. 2015;44(4):1148-60.
14. Wang N et al. The effect of maternal prenatal smoking and alcohol consumption on the placenta-to-birth weight ratio. Placenta. 2014;35(7):437-41.
15. Greenland S, Mansournia MA, Altman DG. Sparse data bias: a problem hiding in plain sight. Bmj. 2016;352:i1981.
16. Gosho M, Ohigashi T, Nagashima K, Ito Y, Maruo K. Bias in Odds Ratios From Logistic Regression Methods With Sparse Data Sets. J Epidemiol. 2023;33(6):265-75.
