# Supplementary material for: Prenatal and birth factors associated with child autism diagnosis: a birth cohort perspective
Source: Pediatr Res. 2026 Jan 15;100(3):1129–42. doi: 10.1038/s41390-025-04747-x (PMC13672113; doi:10.1038/s41390-025-04747-x)
Supplement: Supplementary file 5 [file 41390_2025_4747_MOESM6_ESM.pdf]

Supplementary results:

Sparse bias analysis

To further address potential sparse bias in six of our logistic regression models, we conducted a sensitivity analysis where we fit Bayesian penalised logistic regression models with a F (2,2) prior distribution. Compared to the original models, the odds ratio estimates for autism diagnosis changed by less than 20% for all exposures, except any recreational marijuana use during pregnancy, and remained significant at the alpha level of 0.05 for all exposures except preterm birth (any recreational marijuana use during pregnancy: from 5.68 (95%CI=1.46,22.62;P=0.014) to 4.11 (95%CI 1.05,16.09; P=0.042); single parent during pregnancy: from 3.62 (95%CI=1.29,10.17; P=0.015) to 3.12 (95%CI 1.13-8.63;P=0.028), vinyl flooring in living areas during pregnancy: from 4.21 (95%CI=1.45,12.21; P=0.008) to 3.54 (95% 1.24,10.13; P=0.018); SSRI/SNRI use during 1st and 2nd trimesters: from 6.43 (95%CI=2.76,14.98; P<0.001) to 5.63 (95%CI=2.43,13.06;P<0.001); SSRI/SNRI use during 3rd trimester: from 9.01 (95%CI=3.70,21.9; P<0.001) to 7.67 (95%CI=3.16,18.58; P<0.001); preterm birth (32-37 weeks vs 38 weeks or more): from 2.58 (95%CI=1.02,6.51; P=0.044) to 2.36 (95%CI=0.95,5.82; P=0.060)). Therefore, a low prevalence did not appear to explain these findings

Supplementation during pregnancy

We also assessed supplementation of key nutrients and minerals (Table S6). There was evidence that maternal supplementation with folic acid during pregnancy was associated with reduced odds of autism (OR=0.61; 95% CI=0.35, 1.07; P=0.083). Similarly, higher ingested folate and folic acid (≥0.376g/day) was associated with reduced odds of autism (OR=0.54; 95%CI=0.30,0.96; P=0.036) compared to bottom quartile (<0.365g/day). Similar patterns were observed for ingested zinc (OR=0.71 per 100mg/day increase; 95%CI=0.51,0.99; P=0.047) and magnesium (OR=0.72 per 100mg/day increase; 95%CI=0.54,0.96; P=0.028). Prenatal folate and folic acid intake was also examined using quartiles (STable7).
