# Supplementary material for: Prenatal and birth factors associated with child autism diagnosis: a birth cohort perspective
Source: Pediatr Res. 2026 Jan 15;100(3):1129–42. doi: 10.1038/s41390-025-04747-x (PMC13672113; doi:10.1038/s41390-025-04747-x)
Supplement: Supplementary file 6 [file 41390_2025_4747_MOESM7_ESM.pdf]

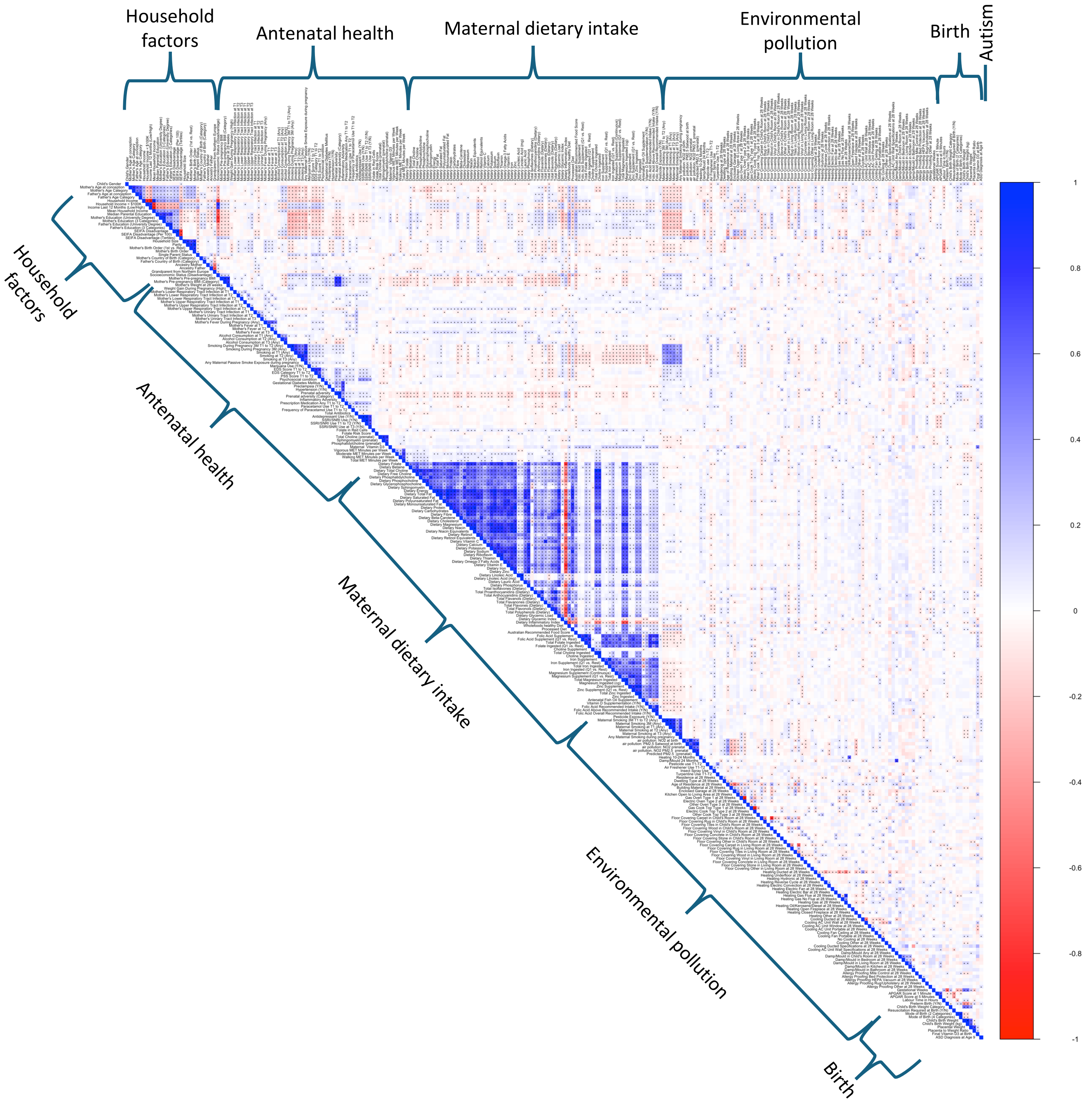

Figure S1 Correlation matrix of all prenatal and birth factors explored in this study including multiple groupings and cut-offs for categorisation of variables. The correlation matrix is depicting the interplay amongst factors of interest including directionality red – positive correlation, blue- negative correlation.
