# Supplementary material for: Prenatal and birth factors associated with child autism diagnosis: a birth cohort perspective
Source: Pediatr Res. 2026 Jan 15;100(3):1129–42. doi: 10.1038/s41390-025-04747-x (PMC13672113; doi:10.1038/s41390-025-04747-x)
Supplement: Supplementary file 7 [file 41390_2025_4747_MOESM8_ESM.pdf]

Supplementary methods:

Box S1 Summary list of all factors including household and demographic composition, maternal health, dietary intake and supplementation during pregnancy, and environmental factors and pollution

Household and demographic composition

Mother's age at conception, father's age at conception, child sex assigned at birth, household income, mother university educated, father university educated, Socioeconomic Indexes for area (SEIFA)[1], Household size, parity, birth order, single parent, all grandparents are of European descent, socioeconomic disadvantage

Maternal health

Maternal pre-pregnancy BMI (kg/m2), maternal infections during pregnancy, fever during pregnancy, any alcohol intake during pregnancy, any smoking during pregnancy, any recreational marijuana drug use, Edinburgh Postnatal Depression Scale [4], Perceived stress score[5], pre-existing psychosocial conditions, gestational diabetes mellitus, preeclampsia, hypertension, weight gain during pregnancy, prenatal adversity score, any prescription medication use during pregnancy, paracetamol use during pregnancy, antibiotic use during pregnancy, SSRI/SNRI use during pregnancy, physical activity

Dietary intake and supplementation during pregnancy

folate, betaine, total choline, free choline, phosphatidylcholine, phosphocholine, glycerophosphocholine, sphingomyelin, energy, all fat, saturated fat, polysaturated fat, monosaturated fat, protein, carbohydrate, fibre, beta-carotene, cholesterol, magnesium, niacin, retinol, vitamin C, calcium, potassium, sodium, riboflavin, thiamine, omega 3, vitamin E, iron, zinc, linoleic acid, lauric acid, phosphorus, isoflavones, proanthocyanin, anthocyanidins, flavanols, flavanones, flavones, flavonols, total polyphenols, glycaemic load, glycaemic index, dietary inflammatory index, wholefoods healthy diet, processed diet, ultra processed food, Australian Recommended Food Score, folic acid supplementation, choline supplementation, iron supplementation, magnesium supplementation, zinc supplementation, fish oil supplementation, vitamin D supplementation

Environmental factors and pollution

Pesticide use during pregnancy, passive tobacco smoke during pregnancy, ambient air pollution, pain use in pregnancy, air freshener use during pregnancy, insect spray use during pregnancy, turpentine use during pregnancy, residence type during pregnancy, age of residence during pregnancy, residence material during pregnancy, oven type use during pregnancy, flooring in child's room, living room during pregnancy, heating and cooling in residence during pregnancy, signs of mould and/dampness, allergy proof methods

Birth factors

Gestational age at birth, Apgar score, labour duration, preterm birth, birth weight, resuscitation required at birth, mode of delivery, child weight, placental weight, placenta-to-birth weight ratio
