# Supplementary material for: Prenatal and birth factors associated with child autism diagnosis: a birth cohort perspective
Source: Pediatr Res. 2026 Jan 15;100(3):1129–42. doi: 10.1038/s41390-025-04747-x (PMC13672113; doi:10.1038/s41390-025-04747-x)
Supplement: Supplementary file 8 [file 41390_2025_4747_MOESM9_ESM.pdf]

Box S2 Generating literature network (Figure 3)using VOSViewer and literature search in Web of Science

We employed literature network visualisation using VOSviewer version 1.6.20 [2]. Same literature network analysis has been used to explore physiological mediators of prenatal environment in autism, which has been previously published [3]. This method measures the strength of links between published literature by examining keywords associated with the articles and then groups these links into non-overlapping clusters. In summary, we have used the following searches using the Web of Science database for each of the five key exposures 1) *socioeconomic status* ((“SES” OR “socioeconomic” OR "socioeconomic status") AND (“inflammation” OR "oxidative stress") AND “pregnancy”)) with N=189 papers, 2) *mental health* ((“depression” OR “anxiety” OR "mental health") AND (“inflammation” OR "oxidative stress") AND “pregnancy”)) with N=680 papers 3) *obesity* ((“obesity”) AND (“inflammation” OR "oxidative stress") AND “pregnancy” AND “human”)) with N=333 4) *diet and nutrition* ((“diet” OR “folate” OR "folic acid" OR “iron“ OR “zinc” OR “magnesium”) AND (“inflammation” OR "oxidative stress") AND “pregnancy”)) with N=1249 papers and 5) *environmental pollution* (("ambient pollution" OR "environmental pollutants" OR "chemicals" OR "smoking") AND (“inflammation” OR "oxidative stress") AND “pregnancy”)) with N=429 papers. General words shared across the search terms, as well as words related to techniques and specific methods, were deselected to better represent the relationships among key biological processes of interest. The default cluster settings were used for consistency. Clusters that were identified were scaled based on the total link strength. Network visualisation was used as final presentation (Figure 3).
